# Prevalence of technology facilitated and other gender-based violence among adolescent girls in Gqeberha, South Africa and its association with probable common mental disorders

**DOI:** 10.3389/fgwh.2025.1546901

**Published:** 2025-09-09

**Authors:** Miriam Aviva Hartmann, Erica Browne, Shepherd Mutangabende, Patience Mungwari, Danielle Stotesbury, Nataly Woollett, Anna Kågesten, Sarah T. Roberts, Abbey Hatcher

**Affiliations:** 1 Women's Global Health Imperative, RTI International, Berkeley, CA, United States; 2 Department of Global Public Health, Karolinska Institutet, Stockholm, Sweden; 3 No Means No South Africa, Gqeberha, South Africa; 4 No Means No Worldwide, McLean, VA, United States; 5 School of Public Health, University of the Witwatersrand, Johannesburg, South Africa; 6 Department of Visual Arts, University of Johannesburg, Johannesburg, South Africa; 7 Department of Health Behavior, University of North Carolina, Chapel Hill, NC, United States

**Keywords:** technology-facilitated violence, gender-based violence, adolescent health, mental health, South Africa

## Abstract

**Background:**

Emerging evidence is raising alarms that technology facilitated gender-based violence (TF-GBV) is a growing public health concern with impacts on child wellbeing, yet little research on the topic has been conducted in middle-income country settings. This study aimed to investigate the prevalence of TF-GBV, other GBV, and their association with common mental disorder (CMD) symptoms among adolescent girls in South Africa.

**Methods:**

Trained enumerators facilitated surveys on exposure to physical and sexual violence with adolescent girls aged 10–19 from 14 low-income primary and secondary public schools. An index of TF-GBV assessed past-year exposure to acts such as public posting of sexual photos. CMD screening used Patient Health Questionnaire-2 and Generalized Anxiety Disorder-2 tools. Generalized estimating equations assessed associations between violence (TF-GBV, other GBV, or both) and CMD.

**Results:**

A total of 1,540 adolescent girls participated in the study. Most participants identified as Black (84%). CMD symptoms were more prevalent among girls in secondary school (37%) than primary school (10%). All forms of past-year GBV were more prevalent among secondary school girls, including TF-GBV (43% vs. 11% in primary school girls). Exposure to both TF-GBV and other forms of GBV were significantly associated with a 3.68 times higher risk of CMD (aRR = 3.68, 95% CI 2.42–5.62) after adjusting for demographics and partnership status.

**Conclusion:**

These findings underscore the need for targeted content to address TF-GBV within existing GBV programs, and its impact on mental health among adolescent girls in similar contexts.

## Introduction

Emerging evidence is raising alarms that technology facilitated gender-based violence (TF-GBV) directed towards adolescent girls and young women is a public health concern of significance. Defined as any harmful act that is assisted, aggravated or amplified by the use of digital technologies and based on inequitable gender norms (1), TF-GBV has been found to impact anywhere from 16% to 58% of adolescent girls and women globally (2). The early age at which these experiences occur is also concerning. In a global study of 22 countries, a majority of young women aged 15–25 years with prior exposure to TF-GBV reported that they first experienced this type of violence before reaching the age of 15 (3).

Although little research measuring the drivers or health impacts of TF-GBV has been conducted, it is thought to be driven by gender norms and power inequalities related to gender, sexuality, race, and disability status that contribute to other experiences of GBV (4, 5). An extensive body of evidence exists highlighting the relationship between GBV, more broadly, including its mental, physical, and social impacts (6–9). These are particularly concerning for adolescent girls, whose behavioral, biological, and social development can be severely impacted by violence exposure during a critical life period, as highlighted in a recent review by Jagasia et al. (10). Similar to other forms of GBV, emerging evidence demonstrates significant mental health consequences of TF-GBV across the life course, including on common mental disorders (CMD) (11) —a category that includes conditions such as anxiety (12, 13), depression (12–14) —as well as related symptoms or sequelae such as substance abuse (15), and impaired academic and occupational functioning (15). These risks are likely to be compounded in the South African context, home to Africa's highest digital access rate, including among adolescents (16, 17), and some of the world's highest rates of GBV (18). While the South African government's National Strategic Plan on Gender-Based Violence and Femicide (NSP-GBVF) (18) acknowledges online violence as a growing concern, evidence of its implementation remains scarce. Similarly gaps in prevalence and impact data on TF-GBV among adolescent girls is limited across low- and middle-income country (LMIC) settings where online access among youth is high, including South Africa in particular (12). A recent systematic review of TF-GBV in LMICs found that only two studies quantitatively examined both prevalence and associated mental health outcomes — and both focused on adult women rather than adolescents (19). These gaps limit our ability to develop age- and context-appropriate prevention strategies.

Improved prevalence data on exposure to TF-GBV among adolescents in LMICs would not only broaden our understanding of this phenomenon in diverse settings, but also inform prevention efforts, which are crucial for healthy individual and societal development. This cross-sectional exploratory analysis among primary and secondary school adolescent girls, ages 10–19, in urban South Africa aims to fill existing research gap. It sheds light on the prevalence of TF-GBV exposure, its intersection with other forms of GBV (henceforth referred to as “other GBV”), and their association with CMD symptomology. Results seek to guide future research and intervention work in this arena.

## Methods

### Overall study design

Data were drawn from the baseline survey of the *No Means No (NMN)* evaluation study, a cluster-randomized control trial (cRCT) conducted in Gqeberha, South Africa. The primary aim of the overall study is to test the effectiveness of the No Means No (NMN) GBV prevention intervention on adolescent girls' reported experiences of sexual violence. The trial enrolled 1,540 adolescent girls, ages 10–19, from 15 low-income primary and secondary public schools randomized in 2:1 to either the intervention or the control (Life skills curriculum, delivered by schools). A list of all schools in the Gqeberha school district was used as our sampling frame, from which we eliminated schools that were outside of the central city. Schools were then stratified by school type (primary vs. secondary) and primary language of instruction (Afrikaans vs. isiXhosa). Five sets of 3 schools were selected from the same strata, based on location, ensuring geographical separation and reduce potential contamination and to enhance feasibility. Participants were followed for 12 months with surveys conducted at baseline, month 3, 6, and 12. The trial is registered on https://www.clinicaltrials.gov (NCT05295342) and results are presented elsewhere (20).

### Study setting

Gqeberha, formerly Port Elizabeth, is the largest city in the Eastern Cape Province, South Africa, and part of the Nelson Mandela Bay Metropolitan area. When compared to other regions, Nelson Mandela Bay Metropolitan area accounts for a total population of 1.26 million, or 18.0% of the total population in Eastern Cape Province. Known as “the friendly city”, it is located on the Indian ocean and is home to the only international airport in the Eastern Cape and two shipping ports. While it contributes the highest proportion of gross domestic product in the Eastern Cape, it also suffers from a high proportion of people living in poverty, at 38.7% in 2016, and unemployment is high (36.6%) (21, 22). It also has the second highest overall crime index of the sub-regions within the province, including a notably high rate of sexual crimes at 242 per 100,000 people (21).

South Africa guarantees universal access to primary and secondary education, however their performance is sharply divided along public and private lines and largely continues a legacy of apartheid era segregation (23). The majority of children in the country attend no-fee government schools, serving low-income communities (24). In the Eastern Cape, public schools disproportionately serve Black South African youth, and the province routinely reports some of the lowest educational outcomes in the country, driven by poorly maintained schools, teacher absence, and lack of school safety (25). Public schools in Gqeberha are co-educational, and while gender-based school segregation is not practiced, schools often reflect and reinforce broader structural gender inequalities in their staffing, curricula, and informal student dynamics. These dynamics are compounded by South Africa's high income inequality (Gini coefficient: 0.63) (26) and persistent gender inequality, which shape adolescents' everyday experiences and opportunities both inside and outside of the classroom (27).

### Participant recruitment and procedures

Recruitment for the baseline survey occurred within 14 of 15 schools randomized to take part in the NMN cRCT from February to October 2022. One school chose not to participate in the study. Within each school, the research team worked with administrators to select classrooms to enroll. For primary schools, these included grade 5–6 classrooms. For secondary schools, classrooms from grades 8–10 were included. Participants were sensitized to the study at the classroom-level and verified for eligibility, including being aged 10–19, not planning to move schools during the study period, willing to participate in the intervention, and English, isiXhosa, or Afrikaans speaking. Participants signed either an informed consent, if 18 years of age or older, or an assent alongside parental consent, if under 18. All survey instruments were translated and back-translated from English into isiXhosa and Afrikaans using a standardized process to ensure linguistic and conceptual equivalence. Translations were reviewed by bilingual research staff with experience in gender and adolescent health research. Surveys were read aloud in the classroom by trained enumerators fluent in all three languages, while participants self-administered the paper-based survey. Participants were able to ask for clarification in their preferred language during administration. Although most participants chose to use the English version, language usage was not systematically recorded. Survey responses were entered into the secure web-based platform REDcap (Research Electronic Data Capture) (28) by Research Assistants using a double-entry system for quality control. The survey took on average 30–45 min to complete and assessed baseline sociodemographic characteristics, as well as exposure to TF-GBV and other GBV.

### Measures

#### Outcome variable

Mental health was measured using two brief screening tools for self-reported symptoms of depression and anxiety, previously validated in South Africa in English and isiZulu (29). These were selected to reduce participant burden and enhance feasibility among school students. *Depression symptomology* was measured using the 2-item Patient Health Questionnaire (PHQ-2). The PHQ-2 scores range from 0 to 6. Depression symptomology was dichotomized to represent the presence of probable clinical depression as defined as a score of 3 and above (0 = no, 1 = yes). *Anxiety symptomology* was measured using the 2-item Generalized Anxiety Disorder (GAD-2), whereby scores range from 0 to 6 with scores of 3 and above indicating a probable anxiety disorder. Anxiety symptomology was dichotomized to represent the presence of probable anxiety disorder (0 = no, 1 = yes). Presence of either depression or anxiety disorder symptoms were further combined into the variable *probable common mental disorder* where an indication of either probable depression or probable anxiety was classified as the presence of probable common mental disorder (CMD) (0 = no, 1 = yes). This combined outcome measure is consistent with standard practice in mental health research, where depression and anxiety are often considered together due to their high comorbidity and common underlying risk factors (11). Additionally, using probable CMD as a single outcome preserved statistical power and aligned with the broader focus of mental health interventions, which typically address both conditions simultaneously (30). Internal consistency in our sample was alpha = 0.79 for the PHQ-2 and alpha = 0.66 for the GAD-2, which is similar to those reported in prior South African validation work (PHQ-2: alpha = 0.71; GAD-2: alpha = 0.62) alongside acceptable classification accuracy (PHQ-2 AUC = 0.72; GAD-2 AUC = 0.69) (29).

#### Exposure variables

The scale used to measure TF-GBV consisted of nine-items from two sub-scales: one for intimate partner violence-related TF-GBV (binary items) and one for TF-GBV committed by an unspecified perpetrator (frequency-based items). Given the limited availability of validated scales for TF-GBV in South Africa, this study used a measure that was developed and validated among adolescents in the US. It was pre- and pilot-tested, along with all survey items, among South African adolescent girls from primary and secondary schools in this setting for face validity. Content validity was assessed through exploratory factor analysis. Reliability analyses indicated acceptable internal consistency for the sub-scales (alpha = 0.75 for the IPV sub-scale, alpha = 0.67 for the non-partner sub-scale). For the combined scale, alpha was 0.77 reflecting acceptable reliability for exploratory research. TF-GBV items asked participants how often in the past year either a partner or someone else did specific behaviors to them, e.g., publicly posting a naked or sexual photo, pretending to be another person online to test them. Items asking about perpetrator-agnostic exposures had four response options for frequency: “never”, “a few times”, “once or twice a week”, or “every day or almost every day”. Those asking about partner-enacted behaviors were “yes” or “no.” A binary variable was created where reports of any frequency of exposure more than “never” was categorized as experiencing that behavior. These were further combined into a binary variable indicating whether someone had experienced any TF-GBV in the past year (0 = no, 1 = yes).

Other forms of GBV were measured using 18 items asking about sexual violence including sexual harassment perpetrated by partners or non-partners, and physical intimate partner violence (IPV). Measures of sexual violence included five items measuring sexual harassment, such as having sexual rumors spread about oneself, being touched in a sexual way, or being forced to kiss someone in the past 12 months; and four items on forced or coerced sex by a partner or non-partner in the past 12 months. Incapacitated sex was measured using two items assessing whether someone had done something to the participant when they were too drunk or high to stop them, or gave them alcohol or drugs to do something sexual with them. Finally, physical IPV was assessed using seven items inquiring about threats or use of physically violent behaviors such as slapping, pushing, hitting, kicking, or choking in the past 12 months. All GBV questions were adapted from those previously used elsewhere for the measurement or evaluation of violence prevention interventions among adolescents (31–33). A binary variable was created to indicate whether someone had experienced any other GBV in the past-year (0 = no, 1 = yes).

We further created a categorial variable including mutually exclusive categories of violence exposure (i.e., TF-GBV only, other GBV only, and experience of both TF-GBV and other GBV). Experience of no violence was the reference category.

#### Covariates

Demographic covariates included participants' self-reported age, race (Black, Colored, Indian, White, or Other), and number of people in the household (1–4, 5–8, and 9 and above). Whether the participant ever had an intimate partner at baseline was also included given its potential association with exposure to violence.

### Analysis

Prevalence estimates were calculated for the outcome (CMD), exposure (TF-GBV, other GBV, or both) and covariate (demographics) variables. Differences in these variables by level of schooling (primary vs. secondary) were investigated using *t*-tests for continuous variables (e.g., age) and chi-square tests for most binary and categorical variables.

Associations between the outcome variable—probable CMD—and the categorical combined variable of GBV exposure were explored using generalized estimating equations (GEE). GEE models offer more robust standard errors even when correlation structures are misclassified (34). The xtgeebcv command with bias-corrected variances was used with a Poisson regression model clustered by school with a log link function and exchangeable correlation (35). Although probable CMD is a binary outcome, Poisson regression with robust standard errors was used to estimate risk ratios, as this approach is widely recommended for binary outcomes when risk ratios are preferred over odds ratios and log-binomial models may not converge (36). The Fay and Graubard standard error correction was used due to unequal/high variability in cluster sizes (37). The model was adjusted using age, ethnicity, number of people in the household, and whether the participant ever had an intimate partner at baseline. Following this, we changed the reference variable in the categorical exposure variable to other GBV-only such that we could compare the strength of association with CMD between (i) exposure to TF-GBV and (ii) both TF-GBV and other GBV, compared to other GBV-only. All analyses were conducted on the full sample. Missingness of responses to individual sub-items for outcome and exposure variables was investigated. Responses to items on forced or coerced sexual violence or incapacitated sex had the greatest number missing with 87% of participants responding to all 6-items and 96% responding to at least 3-items. All other sub-categories had response levels of 95% and above. Within the specific missing items, no clear patterns of missingness were identified. Based on this, missing responses to individual items were coded as non-exposure and individuals were only missing an outcome or exposure variable if they skipped all questions included in that variable. Missingness across the coded outcome and exposure variables and covariates was less than one percent. STATA v 17 (38) (StataCorp 2021, College Station, TX) was used for all analyses with significance at the alpha = 0.05 level.

### Ethical considerations

The study was reviewed and approved by the Human Sciences Research Council in South Africa (REC 2/17/03/21) and received approval for analysis of data from the Swedish Ethical Review Authority (Dnr 2022-03745-01). All participants provided written informed consent or assent (if under 18 years) prior to enrollment. Numerous other considerations were taken given the study's focus on GBV and enrollment of minors following WHO recommendations on conducting research on violence against women and girls (39, 40). The research team was trained on ethical and safety guidelines for conducting such research and followed a standardized protocol for responding to reports of GBV using the LIVES approach (41) and reporting these cases according to the Children's Act of 2005 in South Africa (42). Trained social workers, who were part of the study team, responded to all reported cases of GBV and reports of probable CMD.

## Results

### Participant characteristics

Participants were on average 13 years old, majority Black (82.7%), and isiXhosa speaking (79%), and lived with an average of 6 people in their household. Just over half reported ever having an intimate partner (52%). However, significantly more secondary school adolescent girls compared to primary school adolescent girls ever had a partner (72% vs. 23%) and currently had a partner (42% vs. 21%). See Table 1.

**Table 1:** Participant baseline characteristics, overall and by school level.

Factor	Total	Primary	Secondary	*p*-value
*N* (%)	1,454 (100%)	600 (41.3%)	854 (58.7%)	
Age mean (SD)	13.1 (2.1)	11.0 (1.1)	14.5 (1.1)	<0.001
Race/ethnicity				
Black	1,203 (82.7%)	512 (85.3%)	691 (80.9%)	<0.001
Colored	198 (13.6%)	57 (9.5%)	141 (16.5%)	
Indian	5 (0.3%)	5 (0.8%)	0 (0.0%)	
White	13 (0.9%)	9 (1.5%)	4 (0.5%)	
Language spoken at home				
Afrikaans	144 (9.9%)	46 (7.7%)	98 (11.5%)	0.013
IsiXhosa	1,149 (79.0%)	497 (82.8%)	652 (76.3%)	
English	97 (6.7%)	34 (5.7%)	63 (7.4%)	
Ever had an intimate partner	756 (52.0%)	139 (23.2%)	617 (72.2%)	<0.001
Currently have an intimate partner	490 (33.7%)	127 (21.2%)	363 (42.5%)	<0.001
Number of people in household, mean (SD)	6.2 (4.7)	6.9 (5.8)	5.8 (3.6)	<0.001

SD, standard deviation. 7 participants reported “other” as their race/ethnicity. 28 were missing responses. 14 participants reported “other” as their language spoken at home, 50 were missing responses. 12 participants were missing a response to whether they ever had an intimate partner. 11 participants were missing responses to whether they currently had an intimate partner.

### Mental health status

The mental health status of participants varied significantly by school level with 37.1% of secondary school participants reporting probable CMD compared to approximately 10% of primary school students (*p* < 0.001). The proportion of participants reporting symptoms of depression was equivalent to those reporting symptoms of anxiety (i.e., 19% for both). See Table 2. Mean scores on the PHQ-2 and GAD-2 were 1.0 (SD = 1.7) and 1.1 (SD = 1.7), respectively, indicating an overall low level of symptom burden in the full sample, despite a notable proportion of participants meeting criteria for probable anxiety or depressive disorders.

**Table 2:** Participant probable mental health status, overall and by school level.

Mental health	Total	Primary	Secondary	*p*-value
*N* (%)	1,454 (100%)	600 (41.3%)	854 (58.7%)	
Probable anxiety disorder (GAD-2)	288 (19.9%)	33 (5.5%)	255 (29.9%)	<0.001
Probable depression disorder (PHQ-2)	273 (19.0%)	40 (6.8%)	233 (27.4%)	<0.001
Probable common mental disorder	372 (25.8%)	57 (9.7%)	315 (37.1%)	<0.001

Mental health measures indicate probable mental health disorders based on non-clinical screening. Scores ≥3 on GAD-2 indicate symptoms of generalized anxiety. Scores on PHQ-2 range from 0 to 6 with scores ≥3 indicating probable symptoms of major depression. Probable common mental disorder (CMD) refers to the presence of either clinically relevant depression or anxiety symptoms.

### Prevalence of exposure to TF-GBV and other forms of GBV

The overall prevalence of TF-GBV was 29% (secondary school: 42%; primary school: 11%) with the most common experience being having someone try to get them to talk about sex when they did not want to. Overall, 12.7% of participants reported this occurring a few times in the past year. This was followed by being asked to do something sexual over technology (7.2% experienced a few times), and subsequently by being sent a naked or sexual picture or video (6.6% experienced a few times). Few participants reported these exposures occurring weekly or daily. The most common partner-based TF-GBV exposures were having a partner check one's mobile phone or social media account without permission (12.0%) and having a partner log into their social media account without permission (11.5%). Most TF-GBV exposures were significantly more likely to be reported by secondary school students than primary school students. See Table 3.

**Table 3:** Participant exposure to technology-facilitated gender-based violence and other gender-based violence, overall and by school level.

Forms of TF-GBV and other GBV	Total	Primary	Secondary	Global *p*-value
*N* (%)	1,454 (100%)	600 (41.3%)	854 (58.7%)	
Specific TF-GBV item^a^				
Someone tried to get them to talk about sex				<0.001
A few times	184 (12.7%)	20 (3.4%)	164 (19.3%)	
Once or twice a week	17 (1.2%)	2 (0.3%)	15 (1.8%)	
Everyday or almost everyday	14 (1.0%)	4 (0.7%)	10 (1.2%)	
Someone asked them to do something sexual				<0.001
A few times	104 (7.2%)	16 (2.7%)	88 (10.4%)	
Once or twice a week	8 (0.6%)	2 (0.3%)	6 (0.7%)	
Everyday or almost everyday	8 (0.6%)	3 (0.5%)	5 (0.6%)	
Someone posted or publicly shared picture or video of them naked or doing something sexual				0.96
A few times	13 (0.9%)	6 (1.0%)	7 (0.8%)	
Once or twice a week	2 (0.1%)	1 (0.2%)	1 (0.1%)	
Everyday or almost everyday	2 (0.1%)	1 (0.2%)	1 (0.1%)	
Someone sent a naked or sexual picture or video of themselves				<0.001
A few times	95 (6.6%)	11 (1.9%)	84 (9.9%)	
Once or twice a week	7 (0.5%)	1 (0.2%)	6 (0.7%)	
Everyday or almost everyday	9 (0.6%)	4 (0.7%)	5 (0.6%)	
Partner sent posts or messages that threatened to destroy property or physically harm	39 (2.7%)	13 (2.2%)	26 (3.0%)	0.32
Partner checked mobile phone or social media accounts without permission	174 (12.0%)	20 (3.4%)	154 (18.0%)	<0.001
Partner logged into social media accounts without permission	167 (11.5%)	18 (3.0%)	149 (17.5%)	<0.001
Partner used technologies to control where they are or who they are with	49 (3.4%)	17 (2.9%)	32 (3.8%)	0.35
Partner pretended to be another person online to test them	162 (11.2%)	22 (3.7%)	140 (16.4%)	<0.001
Form of GBV				
Only TF-GBV	51 (3.5%)	11 (1.8%)	40 (4.7%)	<0.001
Other GBV	200 (13.8%)	71 (11.8%)	129 (15.1%)	
Experienced both other forms and TF-GBV	372 (25.6%)	53 (8.8%)	319 (37.4%)	
No violence exposure	818 (56.3%)	459 (76.5%)	359 (42.0%)	

GBV, gender-based violence. Other GBV includes measures of in-person sexual harassment, forced or coerced sex, incapacitated sex, and physical intimate partner violence. TF-GBV, technology-facilitated gender-based violence and includes measures of sexual harassment occurring online or over the phone by a partner or non-partner. 13 participants were missing responses to all GBV questions.

a All TF-GBV items were asked in relation to experiences occurring over mobile apps, social networks, texts, or other digital communication.

Roughly half of participants overall did not report experiencing any form of GBV (56.3%), however, the largest proportion of the remaining 43.7% of participants were exposed to both other and TF-GBV (25.6%). This was followed by exposure to other GBV-only (13.8%), and lastly by TF-GBV-only (3.5%). Exposure to all categories of violence was higher among those in secondary school (*p* < 0.001) where close to 40% of adolescent girls reported experiencing both other and TF-GBV compared to approximately 9% of adolescent girls in primary school.

### Associations with probable common mental disorders (CMD)

Table 4 presents the results of the GEE Poisson regression for modelling the association between exposure to GBV (ref: no GBV) and symptoms of CMD. In this model, the risk of symptoms of CMD increased progressively based on different GBV exposures. Exposure to TF-GBV only was associated with 1.37 times the risk of CMD (*p* = 0.268) compared to those with no violence. Exposure to other GBV-only was associated with 2.98 times the risk of probable CMD (*p* < 0.001), and experience of both other and TF-GBV was associated with 3.68 times the risk of CMD (*p* < 0.001) compared to no violence exposure. When the reference was changed to other GBV-only (results not shown in table), exposure to TF-GBV only (without other forms of GBV) was associated with a lower risk of CMD (0.46, *p* = 0.007). Similarly, exposure to both other and TF-GBV was associated with 1.24 times the risk of probable CMD compared to other GBV-only although this was not significant (*p* = 0.133).

**Table 4:** Adjusted risk ratios (RR) of probable common mental disorder (CMD) by type of gender-based violence (GBV) exposure, estimated using poisson GEE models.

Category of GBV	RR	95% CI	*p*-value
Only TF-GBV	1.37	0.76–2.48	0.268
Other GBV	2.98	2.30–3.86	<0.001
Both other and TF-GBV	3.68	2.42–5.62	<0.001
Ref: no violence			

Risk ratios (RR) were estimated using Poisson regression with robust standard errors within a generalized estimating equation (GEE) framework, to account for clustering by study site. Models were adjusted for age, ethnicity, household composition, and partner status. GBV, gender-based violence. Other GBV includes measures of sexual harassment, forced or coerced sex, incapacitated sex, and physical intimate partner violence. TF-GBV, technology-facilitated gender-based violence and includes measures of sexual harassment specifically occurring online or over the phone by a partner or non-partner. Common mental disorder (CMD) symptomology refers to the presence of clinically relevant depression or anxiety symptoms.

## Discussion

This study, which represents one of the first to quantify the association between TF-GBV and CMD symptoms among adolescents in an LMIC context, found that nearly one-third (29%) of in-school girls reported past-year exposure to any type of TF-GBV. One-quarter (26%) of girls experienced both other and TF-GBV concurrently, while a smaller proportion (4%) experienced TF-GBV on its own. The majority of TF-GBV outcomes were higher among secondary school adolescent girls (43% vs. 11% in primary school), although some specific behaviors—such as online threats, image-based abuse, and technology-based control—were reported at similar levels irrespective of grade, albeit low levels (0.9%–3.4%). Exposure to both TF-GBV and other forms of violence, compared to those with no exposure, was associated with significantly higher levels of reported anxiety and depression symptoms (aRR = 3.68, 95% CI 2.42–5.62).

Adolescence represents a particularly critical phase of development where patterns of mental, physical, and sexual health are established that shape future health and wellbeing (43). Much previous literature shows that GBV has an impact on all aspects of wellbeing and that exposure to violence in childhood can have a lasting effect (44, 45). Our research, which suggests that there is important overlap of TF-GBV and other forms of GBV, and that this co-occurrence may exacerbate the impact of GBV on mental health, draws attention to the ways in which adolescent experiences online may magnify – rather than just mirror – the impact of “offline” experiences. This is aligns with theoretical frameworks like the transformation approach, on the integration of adolescent peer experiences in-person and online, which suggest that aspects of the online experience—such as immediacy, permanence, and audience reach—can intensify effects of in-person experiences (46, 47). While the risk of probable CMD was elevated across all GBV exposure types, we note that the risk ratio was lower in the TF-GBV only group than what we see for those exposed to other GBV or both forms. However, the confidence intervals were wide and this was based on a small number of participants (*n* = 51), limiting interpretability. The higher risk observed among those experiencing both TF-GBV and other GBV – relative to those with no exposure–may, in fact, reflect the kind of intensification this framework describes—where online violence amplifies the psychological toll of offline violence. Further research with larger sample sizes is needed to better understand whether and how only experiencing TF-GBV may differentially impacts mental health compared to other and intersecting forms of GBV.

It is possible that TF-GBV exposure may also have impacts on other aspects of adolescent health beyond mental health. While data is lacking on adolescents, we do know that TF-GBV impacts several spheres of adult women's lives in LMICs. These include effects on (a) psychology: anxiety, loss of confidence, and feelings of isolation; (b) social engagement: withdrawal from social spaces; c) behavior: substance use, aggression, and use of violence; and (d) physical health: poor sleep or other somatic symptoms of stress (19). The potential sexual and reproductive health impacts of TF-GBV are also understudied, despite well-established links between “other” (in-person) GBV and unintended pregnancy, HIV/STIs, and pregnancy loss. This association operates through reduced autonomy and agency for health care-seeking, increased sexual risk behavior, limited sexual and reproductive control, increased stress levels, and poor mental health (44). Adolescent girls in South Africa experience alarmingly high rates of many of these outcomes (48, 49), highlighting the need for further research to understand how TF-GBV may contribute to these outcomes.

Our study is limited by several measurement issues that currently plague this area of research in LMICs. For one, the measure of TF-GBV used was developed in a high-income country setting given a lack of measures validated in LMICs (1, 50). While we pre- and pilot-tested the measure for understandability and analyzed reliability coefficients, which indicated acceptable internal consistency for exploratory purposes, scores underscore the need for future work to refine and validate TF-GBV measurement tools in this context. These findings should thus be interpreted with caution, acknowledging that it is possible the measure is missing critical TF-GBV contextually unique exposures and thus underestimating the true prevalence of TF-GBV. There is an urgent need for validated measurement tools to assess TF-GBV and future research should prioritize refining the scale to enhance its psychometric properties in low-resource settings. Additionally, due to time constraints and the broader focus of our work on sexual violence prevention, we were unable to comprehensively measure psychological GBV. While several items related to sexual harassment and TF-GBV may reflect psychologically abusive behaviors (e.g., threatening messages, online humiliation), our study did not include a dedicated psychological violence scale. This limits our ability to fully explore the relationship between psychological violence and mental health outcomes. We were also unable to examine associations between GBV exposure and mental health outcomes by perpetrator type (e.g., partner vs. non-partner) due to a combination of limited power and measurement design. Many of our GBV and TF-GBV items were developed to capture experiences regardless of perpetrator identity, and thus cannot all be reliably categorized by relationship type. This choice was intentional, to reflect the complexity and overlap of violence experiences in adolescence, but it limits our ability to assess perpetrator-specific effects in this analysis. This lack of information limits our ability to guide intervention programming targeting both perpetrators, as well as survivors of this form of violence. Lastly, the measurement of only depression and anxiety and not other CMDs (i.e., post-traumatic stress disorder or substance use disorder), as well as our conduct of our study among school going adolescents may limit its generalizability to other vulnerable adolescents. Research should be done to capture a broader range of CMDs and prevalence at a community level to better understand prevalence and risk factors.

Our findings suggest several avenues for intervention. First, given the overlap between TF-GBV and other forms of GBV and their shared mental health consequences, prevention efforts should be comprehensive and begin early. Intervention should particularly occur before and throughout adolescence, as indicated by the substantial difference in prevalence between primary and secondary school girls. Second, there is a need to train the key adult figures who interact with adolescents — including teachers, healthcare workers, and law enforcement officials — to recognize, prevent, and respond to online forms of violence. For example, evidence from high-income settings indicates that teachers are interested in training to address GBV, including online violence, and that such training may improve recognition and support for adolescents (51). Third, TF-GBV-related distress should be integrated into routine mental health and GBV screening in adolescent health services. Promising evidence-based approaches, such as Problem Management Plus (PM+), have shown effectiveness in addressing common mental health disorders following violence exposure in LMICs and could be adapted for adolescents affected by TF-GBV (52). Strengthening adolescent-responsive systems across these domains will be critical to addressing the multifaceted harms of TF-GBV.

## Conclusion

This study highlights the high prevalence of TF-GBV among adolescent girls in South Africa and its association with symptoms of depression and anxiety. Notably, secondary school girls reported significantly higher exposure than primary school girls (43% vs. 11%), suggesting that early adolescence may be a critical window for universal prevention. Given the overlap between TF-GBV and other forms of GBV, interventions must take an integrated approach rather than treating these experiences in isolation. While most current programming focuses narrowly on online safety, gender- and trauma-informed approaches that reflect adolescents’ lived experiences are urgently needed (53). Finally, the strong associations with CMD symptoms underscore the need to consider TF-GBV in broader adolescent health strategies. As TF-GBV becomes increasingly common, integrating its detection and response into mental health, education, and justice systems will be essential to protecting the well-being of young people in digitally connected settings.

## Data Availability

The raw data supporting the conclusions of this article will be made available by the authors, without undue reservation.
